# Does Affective Theory of Mind Contribute to Proactive Aggression in Boys with Conduct Problems and Psychopathic Tendencies?

**DOI:** 10.1007/s10578-018-0806-8

**Published:** 2018-04-27

**Authors:** Steven M. Gillespie, Mickey T. Kongerslev, Carla Sharp, Sune Bo, Ahmad M. Abu-Akel

**Affiliations:** 1 Psychological Sciences, Institute of Psychology, Health and Society, University of Liverpool, Liverpool, L69 3GB UK; 2 Centre of Excellence on Personality Disorder, Region Zealand Psychiatry, Slagelse, Denmark; 3 Department of Psychology, University of Southern Denmark, Odense, Denmark; 4 Psychiatric Research Unit, Region Zealand, Slagelse, Denmark; 5 Department of Psychology, University of Houston, Houston, USA; 6 Department of Child and Adolescent Psychiatry, Region Zealand, Roskilde, Denmark; 7 Institute of Psychology, University of Lausanne, Lausanne, Switzerland

**Keywords:** Adolescence, Conduct disorder, Psychopathy, Personality disorder, Violence

## Abstract

Adolescent psychopathic tendencies are associated with phenotypic increases in proactive aggression. However, the extent to which an understanding of others’ affective mental states, or affective theory of mind (ToM), contributes to proactive aggression remains unknown. We examined how performance on a well-known test of affective ToM, based on cropped images of the eye region, contributes to reactive and proactive types of aggression in a mixed ethnicity sample of 80 incarcerated adolescent boys. A hierarchical regression model showed that affective ToM predicted proactive aggression over and above the influence of clinically rated psychopathic tendencies. Importantly, affective ToM was unrelated to reactive aggression. Our results suggest that being able to recognize others’ affective mental states may be an important factor in aggressing against others for personal gain. These findings have implications for interventions designed to enhance ToM in youth with conduct problems.

Youth with conduct problems (CP) and aggressive, antisocial behavior are a heterogeneous group that incur a considerable societal burden in terms of victimization and financial costs [1, 2]. A particular subgroup of youth with CP also present with elevated psychopathic tendencies, including empathic deficits and a callous and manipulative interpersonal style [3]. These characteristics are specified as ‘with limited prosocial emotions’ in the most recent version of the *Diagnostic and Statistical Manual of Mental Disorders* [DSM-5] [4]. Youth with these tendencies show a poorer prognosis and treatment response [5], are responsible for more varied acts of aggression, more severe harm to the victim, and show increased rates of violent recidivism compared to youth with CP without psychopathic tendencies [1]. In light of these findings, recent CP research has focused heavily on psychopathic tendencies, with the hope that understanding this route will aid the development of more effective programs for prevention and intervention.

Two broad types of aggressive behaviors have been identified in the literature: reactive aggression, also known as impulsive aggression, and proactive aggression, also termed premeditated or instrumental aggression [6–9]. Both types of aggression are increased in relation to psychopathic tendencies [10], but are associated with differing patterns of autonomic and executive functioning [11, 12]. When considering distinct psychopathic traits in adolescents, impulsive psychopathic traits are more strongly related to reactive aggression [13], while psychopathy-linked narcissism appears to be related to increases in both reactive and proactive aggression [14]. More recently, particular attention has been paid to the callous-unemotional (CU) features of psychopathy, and a recent review suggests that high scores on this dimension are associated with increased use of both reactive and proactive aggression, and a more severe and stable pattern of CP [1].

The processes underlying the relationship of psychopathic tendencies with proactive aggression remain poorly understood. One theory suggests that adolescents with elevated psychopathic tendencies show low autonomic arousal, indicated by low resting heart rate, and engage in risk taking and antisocial behaviors as a means of increasing arousal [15]. However, support for this under arousal theory is scarce, and it remains unclear whether low resting heart rate is related to reactive or proactive aggression [16]. Another influential theory suggests that psychopathic tendencies, particularly the CU dimension, are associated with a reduced ability to recognize and experience others distress cues as aversive [5, 17]. It is suggested that these impairments lead to problems in empathic and moral development, and increased use of proactive aggression [5, 17]. In support of this theory, youth and adults with psychopathic tendencies show difficulties in recognizing and processing others facial expressions of emotion [18–21]. Moreover, brain imaging studies suggest that children with CU traits show hypoactivity of the amygdala in response to others fearful expressions [22–25]. Importantly, this pattern of hypoactivity mediates the relationship of CU traits with proactive, but not reactive, aggression [26].

The extent to which theory of mind (ToM) contributes to proactive aggression in youth with CP and psychopathic tendencies remains unclear. ToM, a term first coined by Premack and Woodruff [27], refers to the ability to ascribe feelings, thoughts, and beliefs to others [28]. We have previously highlighted that ToM is a multifaceted construct [29], and research from a clinical neuroscience perspective has defined ToM along various dimensions [30, 31]. One such dimension distinguishes between cognitive ToM, referring to mentalizing about others intentions, beliefs and desires, and affective ToM, referring to mentalizing about others feelings and emotions [32, 33]. Another dimension parses ToM into more bottom-up, automatic ‘mind reading’ mechanisms, and more cognitively demanding top-down processes, required for inferring another’s mental state based on the environment, and previous knowledge about people and events [29, 34, 35].

One aspect of ToM that has been investigated in both adults and developmental samples with elevated psychopathic tendencies is bottom-up, affective ToM. This aspect of ToM has been commonly assessed using the Reading the Mind in the Eyes Test (RMET) [36]. During the RMET, participants are presented with cropped images of the eye region showing complex social emotions and are asked to match the emotion shown in the image to emotion and mental state descriptor words. The relationship of psychopathic tendencies with RMET task performance is somewhat unclear. Some studies suggest that RMET performance may be intact or even enhanced in adult male psychopaths [37, 38]. However, more recent studies in child and adolescent samples have found generally impaired task performance on the RMET in relation to distinct psychopathic traits [29, 39]. For example, in one study of 417 children between the ages of 10 and 12, performance on the RMET was negatively associated with grandiose/manipulative, CU, and impulsive/irresponsible psychopathic tendencies [39]. However, when these dimensions were modelled simultaneously, the CU dimension alone predicted worse performance for complex emotions, but not basic emotions [39]. In a separate study of 342 adolescents recruited from an inpatient psychiatric unit, the CU features of psychopathy were related to worse performance on both the RMET, and the Movie Assessment for Social Cognition [MASC] [40], a more cognitively demanding, top-down measure of ToM [29]. Thus, there is evidence that at least in adolescent samples, psychopathic tendencies are associated with impaired affective ToM. However, the extent to which RMET task performance in youth with CP contributes to proactive aggression remains unknown.

Aggressiveness and affective ToM abilities are also influenced by the presence of personality disorders that are common among youth with conduct problems, including perhaps most notably borderline personality disorder [BPD]. The term BPD refers to a pattern of instability in interpersonal relationships, self-image, and affect, and marked impulsivity [4]. However, despite presenting with marked interpersonal impairments, individuals with BPD often appear to be highly perceptive to others emotional states, and have been found to outperform healthy controls on the RMET [41]. Thus, those with BPD and those with psychopathic tendencies appear to show divergent patterns of performance on tests of affective ToM. Perhaps unsurprisingly, given the pattern of affective instability and impulsivity, BPD is also associated with increased aggressiveness, particularly the reactive type [42, 43]. As such, the presence of BPD, as well as psychopathic tendencies, represents an important variable in trying to understand the relationship of affective ToM with proactive and reactive types of aggression.

In the present study, we aimed to test whether or not the ability to identify others affective mental states, as assessed using the RMET, is related to self-reported reactive and proactive aggression scores in a sample of adolescent males with CP. In particular, we were interested in these relationships after controlling for the presence of other constructs, including clinically assessed psychopathic tendencies and the presence of BPD, that are known to be associated with RMET task performance [29, 39, 41], and with reactive and proactive aggression [1, 5, 42, 43]. Consistent with earlier research, we hypothesized that increasing psychopathic tendencies would be associated with higher levels of both reactive and proactive aggression. Furthermore, because a relatively intact ability to mentalize about others affective states might prove advantageous in manipulating and extorting others for personal gain, we predicted that increased RMET task performance would also be associated with higher levels of proactive aggression. This hypothesis is supported by results from a sample of adult patients with schizophrenia where the ability to understand and reason about others cognitive mental states mediated the relationship of psychopathic traits with proactive aggression [44]. Finally, we predicted that there would be no relationship between RMET task performance and reactive aggression, even after controlling for psychopathic tendencies and the presence of BPD.

## Method

### Participants

The final sample comprised 80 adolescent incarcerated boys, recruited from three secure institutions for juvenile offenders in Denmark (see Table 1 for sample demographic and clinical characteristics). The initial sample consisted of 127 juvenile offenders who were assessed for eligibility. Inclusion criteria were being male, between 15 and 18 years (inclusive; this is the minimum and maximum age range in which minors can be judicially incarcerated in Denmark), remanded (pre- and post-trial) or sentenced, sufficiently fluent in Danish, and willing and able to give informed consent. Forty-seven in total were excluded: 27 refused to participate, 15 did not meet inclusion criteria (four were girls; three were under the age of 15; two were unable to give informed consent due to acutely severe Axis I disorder; and six did not understand Danish sufficiently), and five met exclusion criteria (one was described in files as having severe mental retardation; two were intoxicated on the day of assessment; and two were actively psychotic on the day of assessment).

**Table 1:** Sample characteristics (*N* = 80 boys)

Variable	Category/description	Mean	SD
Age		16.50	0.75
Vocabulary subtest of the WISC-III/WAIS-III^a^		8.51	1.14
Psychopathy Checklist:Youth Version (PCL-YV)		20.58	8.16
Reading the Mind in the Eyes Test (RMET)		23.38	4.07
Reactive-Proactive Aggression Questionnaire			
	Proactive subscale (RPQ-P)	8.19	4.51
	Reactive subscale (RPQ-R)	11.16	6.01

Variable	Category/description	N	%
Ethnicity			
	Immigrant	39	48.75
	Descendant	41	51.25
Present education level			
	In high school	5	6.25
	Technical school apprenticeship	6	7.50
	In elementary school	15	18.75
	Municipal education project	18	22.50
	None	36	45.00
Reason for placement in secure institution			
	Remand	67	83.75
	Sentenced	13	16.25
Previous placement in secure institution		35	43.75
Most severe index offence (ordered by frequency)			
	Robbery (including mugging)	49	61.25
	Assaults	18	22.50
	Theft	6	7.50
	Murder/attempted murder	3	3.75
	Major driving offences	2	2.50
	Sex offences	1	1.25
	Possession of weapons	1	1.25
Personality disorders (SCID-II^b^)			
	Cluster A (paranoid, schizotypal, schizoid)	16	20.00
	Cluster B (histrionic, narcissistic, borderline, antisocial)	52	65.00
	Borderline Personal Disorder	17	21.3
	Cluster C (avoidant, dependant, possessive-compulsive)	2	2.50
Any DSM-IV 10 personality disorders		52	65.00
Mental disorders (K-SADS-PL^c^)			
	Mood disorders	6	7.50
	Schizophrenia and Other Psychotic Disorders	1	1.25
	Anxiety disorders	14	17.50
	ADHD	18	22.50
	Conduct disorder	61	76.25
	Oppositional defiant disorder	31	38.75
	Substance use disorders	46	57.50
	Tic disorders	2	2.50

^a^Proxy scores for verbal IQ were obtained using the vocabulary subtest [56] of the Wechsler Intelligence Scale for Children-Third Edition [WISC-III] [57] in boys < 17 years, and the Wechsler Adult Intelligence Scale-Third Edition [WAIS-III] [58] in boys ≥ 17 years

^b^SCID-II = Structured Clinical Interview for DSM Disorders Axis II Disorders [50]

^c^K-SADS-PL = Schedule for Affective Disorders and Schizophrenia for School age Children-Present and Lifetime Version [55]

### Assessment Instruments

#### Psychopathic Tendencies

The Psychopathy Checklist:Youth Version [PCL:YV] [3] was used for the assessment of psychopathic tendencies. The PCL:YV is a clinical construct rating scale for use with youth aged 12–18, and comprises 20 items tapping the affective, interpersonal, lifestyle, and antisocial features of the disorder. Each item is scored 0 (*the item does not apply*), 1 (*the item applies to some extent*), or 2 (*the item applies to the youth*), on the basis of a semi-structured interview and comprehensive file review. Scores on the 20 items can be summed to yield a dimensional score ranging from 0 to 40. The PCL:YV is one of the most widely used measures of psychopathy in adolescence [45], and has acceptable psychometric properties [46–48]. In this study, interrater agreement on the PCL:YV total scale in a randomly selected subset of our sample (*n* = 20) was excellent (ICC = 0.91) [49].

#### Affective Theory of Mind

The RMET [36], a performance-based measure used to assess participants’ ability to recognize emotions in others based on images of the eye region, was used to assess the recognition of others affective mental states. The test comprises 36 photographs. Participants are asked to make a forced choice as to which word from a choice of four best matches what the person in the photograph is thinking or feeling. For each photograph three of the four words are distractor items, while one of the words represents the correct answer.

#### Reactive Proactive Aggression Questionnaire

The Reactive Proactive Aggression Questionnaire [RPQ] [9] is a 23-item self-report measure made up of two distinct subscales for the assessment of Reactive (11 items) and Proactive (12 items) forms of aggression for use with children and adolescents, and reflects trait characteristics. Each item is scored on a 3-point scale from 0 (*Never*) to 2 (*Often*). Internal consistencies (Chronbach’s alpha) for the RPQ-P (Proactive Aggression) and RPQ-R (Reactive Aggression) in the current sample were 0.85 and 0.93, respectively.

#### Additional Measures

*The Structured Clinical Interview for DSM-IV Axis II Personality Disorders* [SCID-II] [50] was used to asses for personality disorder. The SCID-II is a semi-structured interview consisting of 119 sets of questions—plus some additional questions to assess antisocial personality disorder and conduct disorder prior to age 15. The questions correspond to diagnostic criteria for the respective DSM-IV personality disorders, and are scored as either 1 = absent; 2 = subthreshold; 3 = true or ‘?’ = inadequate information. The SCID-II is widely used in research with adolescents, and has acceptable psychometric properties [51–53]. Interrater agreement in a randomly selected subset of the sample (*n* = 40) was excellent (kappa = 0.81) for a categorical diagnosis of any personality disorder, and good to excellent (kappa = 0.73–0.91) for the diagnosis of specific personality disorders [54]. The *Schedule for Affective Disorders and Schizophrenia for School age Children-Present and Lifetime Version* [K-SADS-PL] [55] was administered to asses for concurrent psychiatric diagnoses. The K-SADS-PL is a semi-structured interview for assessment of current and past psychopathology in children aged 6–18 years, according to DSM-IV criteria. In this study, we only assessed for current psychopathology, and did not include family members as informants. Interrater agreement in a subset of the sample was excellent, with kappa values for categorical agreement and intraclass correlations (ICC) for dimensional agreement of symptoms ranging from 0.77 to 0.86. Finally, we used the vocabulary subtest [56] of the Wechsler Intelligence Scale for Children-Third Edition [WISC-III] [57] in boys < 17 years, and the Wechsler Adult Intelligence Scale-Third Edition [WAIS-III] [58] in boys ≥ 17 years as a proxy for verbal intelligence. Inter-rater agreement in a subset of the sample (*n* = 20) was excellent (ICC = 0.82) [49].

### Procedure

The second author (MTK) performed all assessments in quiet areas at the secure sites. Prior to the assessments, participants were informed about the study aims and procedures and told that their assessment results would be treated with confidentiality and would not be shared with staff, relatives, or anyone else unless the participants gave special permission for this. We provided all participants with clear instructions for completing the self-report inventories. Participants were told that with the exception of the WISC/WAIS test, there were no preferred or ‘correct’ answers to any of the questions, but that they should try to respond as honestly as possible. To minimize test fatigue, participants were told that they were welcome to ask for a break at any point. The PCL:YV, SCID-II, K-SADS-PL, and WISC-/WAIS assessments were audio recorded to allow for assessment of interrater agreement. When data collection was finished, an experienced clinical psychologist (SB) read through file information and listened to randomly selected recordings of the PCL:YV, SCID-II, K-SADS-PL, and WISC-III/WAIS-III assessments, rating them blind to the original ratings so that interrater agreement could be estimated.

### Analytic Approach

The main analyses were conducted using two multiple linear hierarchical regressions to examine the effect of affective ToM (measured with the RMET) on proactive (RPQ-P; Model 1) and reactive (RPQ-R; Model 2) aggression. Due to the high correlations between RPQ-P and RPQ-R (*r* = 0.62, *p* < 0.001), the shared variance between the subscales was removed, and so we used the respective adjusted scores (i.e., residuals) as the dependent variable. The use of residualized scores means we are better able to isolate the relative contribution of affective ToM to proactive and reactive aggression, recognizing the importance of differentiating these two types of aggression [9]. For each model, we report the F_change_ statistics which reflects the additional amount of variance explained by RMET after accounting for all the other variables in the model: proxy scores for verbal IQ, the presence/absence of BPD, and PCL:YV total score. Research on the underlying factor structure of the psychopathy construct as measured by the PCL:YV is equivocal about a three or four factor model [48, 59, 60], and so we chose to use the PCL:YV total score in the regression models reported here. We also control for the presence of BPD due to its association with violence and prevalence in adolescent offenders [54, 61], and due to evidence suggesting that individuals with features of BPD outperform healthy controls on the RMET [41]. Proxy scores for verbal IQ were controlled for based on specific evidence from a recent meta-analysis suggesting that IQ contributes to performance on the RMET [62]. Because participant age was uncorrelated with either RMET total score, or reactive and proactive aggression, in the interests of parsimony the models presented here do not include age as a control variable. However, it should be noted that the inclusion of age in alternative models did not alter the observed pattern of results.

## Results

Sample characteristics, including participants’ scores on the PCL:YV, RMET, RPQ-P and RPQ-R are summarised in Table 1. About half of the participants (49%) were immigrants or descendants, primarily from the Middle East, Northern Africa, or Europe. Most of the participants were remanded, and the majority of participants had an index offense that included a violent element (e.g., robbery (including mugging), assault, murder/attempted murder), and approximately half had lost contact with the educational system prior to incarceration. As can be seen from Table 1, although the rates of personality disorders and other psychiatric disorders were high, they are nonetheless comparable with those reported in other studies of incarcerated youth [63–66].

First, we examined the inter-correlations of age, scores on the PCL:YV, RPQ-P, RPQ-R, and proxy scores for verbal IQ (see Table 2). Higher PCL:YV total scores were associated with worse performance on the RMET, but increased aggression using the RPQ-P and RPQ-R. As expected, increasing scores on a proxy for verbal IQ were associated with better performance on the RMET. Age was not associated with either RMET task performance, or residualized scores for reactive or proactive aggression.

**Table 2:** Inter-correlations of psychopathic tendencies, reactive and proactive aggression, and verbal IQ (*N* = 80)

	PCL:YV	RMET	Proxy verbal IQ	Age
RMET	− 0.32**			
Proxy verbal IQ	− 0.08	0.32**		
Age	0.09	0.13	0.25*	
Adj. RPQ-P	0.41***	0.02	− 0.03	0.04
Adj. RPQ-R	0.31**	− 0.21	− 0.06	0.03

*p < 0.05, **p < 0.01, ***p < 0.001

*PCL:YV* Psychopathy Checklist:Youth Version, *RMET* Reading the Mind in the Eyes Test total score, *Adj. RPQ-P* Reactive Proactive Aggression Questionnaire-Proactive subscale (adjusted for RPQ-R), *Adj. RPQ-R* Reactive Proactive Aggression Questionnaire-Reactive subscale (adjusted for RPQ-P)

Next, the results of two hierarchical regression models probing the unique variance for proactive aggression adjusted for reactive aggression (Model 1) and reactive aggression adjusted for proactive aggression (Model 2) are summarized in Table 3. Step 1 of Model 1 revealed no significant effect for proxy verbal IQ, but a significant negative effect for the presence of BPD on proactive aggression, explaining 14.3% of the total variance *F*(2, 77) = 6.44, *p* = 0.003, *R*^2^ = 0.143. In Step 2, entering PCL:YV total scores improved the model by explaining an additional 23.7% of the variance *F*_change_(1, 76) = 29.01, *p* < 0.001, *R*^2^_change_ = 0.237, and showed that PCL:YV scores were significantly and positively associated with proactive aggression. In Step 3, entering the RMET scores resulted in further improvement of the model by explaining an additional 3.6% of the total variance *F*_change_(1, 75) = 4.62, *p* = 0.035, *R*^2^_change_ = 0.036, or 8.65% of the total variance explained by the overall model *F*(4, 75) = 13.35, *p* < 0.001, *R*^2^ = 0.416. RMET was significantly and positively associated with the unique variance for proactive aggression adjusted for reactive aggression.

**Table 3:** Summary of hierarchical regression analyses

Predictors	Model 1 Proactive aggression^a^			Model 2 Reactive aggression^b^		
	B	SE	β	B	SE	β
Step 1						
Verbal IQ	− 0.171	0.329	− 0.055	− 0.093	0.413	− 0.022
Borderline personality disorder (yes/no)	− 3.254	0.909	− 0.379**	5.593	1.140	0.488***
Step 2						
Verbal IQ	− 0.075	0.283	− 0.024	− 0.034	0.403	− 0.008
Borderline personality disorder (yes/no)	− 4.043	0.792	− 0.470***	5.111	1.129	0.446***
PCL:YV	0.215	0.040	0.496***	0.132	0.057	0.228*
Step 3						
Verbal IQ	0.272	0.291	− 0.087	0.150	0.422	0.036
Borderline personality disorder (yes/no)	− 4.129	0.775	− 0.480***	5.192	1.123	0.453***
PCL:YV	0.244	0.041	0.562***	0.105	0.060	0.182
RMET	0.184	0.086	0.211*	− 0.172	0.124	− 0.149

Bold values represents standardized coefficients

*PCL:YV* The Psychopathy Checklist: Youth Version, *RMET* Reading the Mind in the Eyes Test

*p < 0.05; **p < 0.01; ***p < 0.001

^a^Proactive aggression (Reactive Proactive Aggression Questionnaire-Proactive subscale) scores are adjusted for reactive aggression (Reactive Proactive Aggression Questionnaire-Reactive subscale)

^b^Reactive aggression (Reactive Proactive Aggression Questionnaire-Reactive subscale) scores are adjusted for proactive aggression (Reactive Proactive Aggression Questionnaire-Proactive subscale)

Step 1 of Model 2 (see Table 3) revealed no significant effect for proxy verbal IQ, but a positive significant effect for the presence of BPD on reactive aggression, explaining 22.1% of the total variance *F*(2, 77) = 12.21, *p* < 0.001, *R*^2^ = 0.221. In Step 2, entering PCL:YV total scores improved the model by explaining an additional 5% of the variance *F*_change_(1, 76) = 5.35, *p* = 0.023, *R*^2^_change_ = 0.050, and showed that PCL:YV scores were significantly and positively associated with reactive aggression. In Step 3, entering the RMET scores did not significantly improve the model *F*_change_(1, 75) = 1.93, *p* = 0.169, *R*^2^_change_ = 0.018.

## Discussion

In the present study, we investigated if affective ToM, as assessed with the RMET, contributed to higher levels of reactive (RPQ-R) and proactive (RPQ-P) aggression, over and above the effects of clinically rated psychopathic tendencies (PCL:YV). Using separate hierarchical multiple linear regression models, we found that after controlling for proxy verbal IQ scores, presence of BPD, and psychopathic tendencies, RMET performance contributed to elevated proactive aggression, but not reactive aggression. The specificity of this effect appears to be consistent with the suggestion that reactive forms of aggression may reflect impulsivity and emotion dysregulation [9, 67], rather than abilities for understanding and recognising others affective states.

For regression models predicting reactive and proactive aggression, we used residualized scores of each type of aggression. This allowed us to isolate the contribution of affective ToM to the unique variance associated with each type of aggression, and has the benefit of helping to obtain a clearer understanding of the etiology of aggression in adolescents [9]. When added to the regression models, we found a positive effect of psychopathic tendencies on both reactive and proactive aggression. These findings are consistent with the results of several studies showing that the presence of elevated psychopathic tendencies denotes a particular subgroup of adolescents who show heightened risk for both reactive and proactive forms of aggression [1, 10, 17]. In contrast, the presence of BPD had diametrical effects on the two types of aggression, with a negative relationship observed with proactive aggression, but a positive relationship with reactive aggression. The finding that BPD is related to higher levels of reactive aggression is perhaps unsurprising given that impulsivity and affective dysregulation are core symptoms of the disorder [42, 43]. Our findings therefore provide further support for models that distinguish between reactive and proactive forms of aggression, and which implicate problems in impulse control and emotion regulation in the reactive type [16, 67]. Finally, when RMET performance was added to the models, its effect was specific to proactive forms of aggression, explaining 8.65% of the total variance explained by the overall model.

The finding that improved RMET performance is associated with increased proactive aggression may appear somewhat surprising. For example, aggression is typically thought to reflect impairments in normal social-affective cognition [17], and a meta-analysis has identified a positive, albeit a weak association between ToM and childhood prosocial behaviour [68]. Nonetheless, others have noted that some children who bully require good social-affective cognition in order to organize and manipulate others, cause harm to the victim, and avoid detection [69, 70]. The results reported here extend these findings and suggest that the ability to recognize others affective mental states contributes to proactive aggression, and may confer an advantage for understanding how to inflict suffering, and manipulate and extort others for personal gain [44]. Intriguingly, comorbid schizophrenia/positive psychotic experiences and psychopathic tendencies are associated with benefits in metalizing abilities in clinical and non-clinical samples [71, 72], and with an increased risk for violence [6]. Thus, it is possible that improved mentalizing abilities also contribute to the elevated rates of proactive aggression observed in comorbid schizophrenia and psychopathy.

As highlighted above, it is possible that increased affective ToM may contribute to acting pro-socially in youth without CP and psychopathic tendencies. In trying to consolidate these findings with those of the present study, one hypothesis is that an ability to understand others affective mental states would contribute to proactive aggression among those who also show problems in emotional resonance, that is, the ability to *feel what another is feeling*, and antisocial personality pathology. Notably, the ability to feel what another is feeling is typically lacking among youth with CP and elevated psychopathic tendencies [73], and these children may benefit from the ability to understand their victims’ mental states without experiencing their feelings and emotions [74]. In support of this account, overlapping yet separate neural networks underpin the abilities to ‘*understand*’ and ‘*feel with*’ another’s emotions [75]. An alternative hypothesis is that children who engage in proactive aggression, and manipulate and extort others for personal gain, may become more adept at understanding how others’ minds work. This view is consistent with a ‘theory building’ account of mentalizing, which suggests that, over time, individuals acquire an understanding of others affective mental states and can use this knowledge to speculate about what others think, feel, and believe [74]. Thus, future research should seek to establish those factors that work with affective ToM to predict an increased tendency toward acting aggressively. Longitudinal studies [76] should also be used to establish the causal processes that unfold over time in the relationship of psychopathic tendencies with affective ToM and aggressive behaviours.

Although psychopathic tendencies and affective ToM both appear to be positively related to proactive aggression, it is interesting that we nonetheless observed a significant negative zero-order relationship between PCL:YV scores and RMET performance. These findings contribute to the considerable debate that surrounds the relationship of psychopathic tendencies with RMET performance in adolescents, and are consistent with two recent studies in community and psychiatric adolescent inpatient samples [29, 39]. Elsewhere we have argued that as children with high levels of psychopathic traits begin to age into adolescence, they learn to compensate for reduced amygdala activation associated with stimuli that are supposed to elicit affective responses [77]. However, this compensation appears to be effective only with basic mental states and becomes increasingly challenged as more complex mental state reasoning is required [29, 39].

We would note that there is some debate in the literature as to whether the RMET represents a test of basic emotional expression recognition, or affective ToM. This debate may be informed by a consideration of brain imaging studies which show that the processes underlying emotion recognition and affective ToM appear to be distinguishable, and that increased activation has been observed in several areas implicated in ToM during RMET task performance, including superior temporal sulcus, and inferior frontal gyrus [78, 79]. Thus, while our findings support a contribution of affective ToM to proactive aggression, it would also be interesting to examine if, and how, recognition accuracy for basic facial expressions of emotion, including fear and sadness, contributes to proactive aggression. It is also worth examining whether or not these relationships vary between adolescent girls and boys. Finally, the results reported here are based on a cross-sectional sample, and so causal relationships cannot be assumed.

Our findings may have implications for the assessment of youth with CP, and the development of interventions aimed at reducing aggression. In relation to assessment, the strength of the association between psychopathic tendencies and aggressive behaviour varies greatly [1], and our results suggest that affective perspective taking may aid the prediction of proactive aggression above and beyond the influence of psychopathic tendencies. The inclusion of such variables in assessment procedures may improve the identification of adolescents who are most in need of treatment, and aid the process of allocating individuals to relevant interventions. In relation to treatment, mentalization-based treatment techniques hold promise for persons diagnosed with co-morbid antisocial personality pathology and BPD for reducing feelings of anger and hostility, and improving interpersonal functioning [80, 81]. However, the success of such techniques among individuals with elevated psychopathic tendencies remains unknown. Indeed, although psychopathy is related to deficits in affective ToM [29, 39, 72], the findings of the current study would contraindicate the use of interventions aiming to enhance mentalizing about others affective states in youth with CP and elevated psychopathic tendencies.

Instead, the challenge in early intervention is to understand how youth with CP can use their perspective taking abilities for more prosocial means. As youth age in to adolescence and adulthood they naturally become more accomplished at understanding others’ affective mental states, and there is a normative increase in prosocial behaviour and a decline in aggressive behaviours [82]. Thus, future research should aim to understand how to prevent youth with CP and psychopathic tendencies from using their affective perspective taking abilities to the detriment of others. Interventions targeting social and affective functioning in youth with CP and psychopathic tendencies may benefit from increasing responsiveness to others distress cues [17]. In support of this approach, evidence suggests that directed empathy increases deliberate vicarious representations among adult males [83]. However, whether such techniques are associated with an increased ability to *feel* what another is feeling, or reductions in proactive aggression, is unclear [84]. The use of such methods as part of intensive and targeted interventions that utilise a reward-oriented approach may result in the most positive outcomes [1].

## Summary

This study investigated the contribution of affective ToM to different types of aggression in a sample of incarcerated adolescent boys with CP. We found that increasing affective ToM abilities contributed to greater levels of proactive, but not reactive, aggression, over and above the influence of clinically rated psychopathic tendencies, presence of BPD, and verbal IQ. Importantly, these models were based on residualized values for each type of aggression controlling for the other, and therefore helped to isolate the effects of affective ToM on the unique variance for proactive aggression. These findings support models that highlight differential mechanisms underlying proactive and reactive forms of aggression, and suggest that the ability to mentalize about others affective states may be associated with greater abilities to understand how to hurt others, and manipulate and extort them for personal gain. The results reported here support the conclusions of others [44] that ToM abilities are an important factor in aggressing against others for instrumental purposes. These findings emphasize the importance of distinguishing between types of aggression, and in considering personality variables and social-cognitive abilities in the assessment and formulation of treatment plans for adolescents with CP.
